# MicroRNA 15a and 16 Regulate Proteostasis in Non‐Small Cell Lung Cancer

**DOI:** 10.1096/fba.2026-00075

**Published:** 2026-05-02

**Authors:** Patrick J. Ryan, Bethany C. Guerra, Peter P. Nghiem, Steven E. Riechman, Mariana Janini Gomes, James D. Fluckey

**Affiliations:** ^1^ Muscle Biology Laboratory, Department of Kinesiology and Sports Management Texas A&M University College Station Texas USA; ^2^ Department of Veterinary Integrative Biosciences, College of Veterinary Medicine and Biomedical Sciences Texas A&M University College Station Texas USA

**Keywords:** anabolism, cancer, metabolism, microRNA, proteostasis

## Abstract

Aberrant anabolic activity is critical to tumor biology; however, much remains to be learned about the regulators of protein anabolism in cancer and how this regulation may affect cancer pathophysiology. MicroRNA (miRNA), a family of small nucleotide regulatory molecules, may serve as a potential source of proteostatic regulation. Here, we examined the ability of two co‐transcribed miRNA species, miR15a and miR16 (jointly described as miR15a/16) to regulate protein handling and pathophysiology in non‐small cell lung cancer (NSCLC). We found that miR15a/16 regulates genes in numerous metabolic and pathological pathways, including those related to protein metabolism. Transfection of cellular models of NSCLC with miR15a/16 mimetics caused reductions in both cell growth and protein synthesis rates. These findings indicate that miR15a/16 acts as regulators of protein anabolism in NSCLC, serving as novel metabolic regulators and potential clinical therapeutic targets for malignant lung cancer.

## Graphical abstract

Overexpression of microRNA15a/16 slows rates of tumor growth and protein synthesis in cultured lung cancer cells. These co‐transcribed miRNAs target a wide array of anabolic and pro‐tumor processes, indicating their central role in regulating lung cancer cellular anabolism.

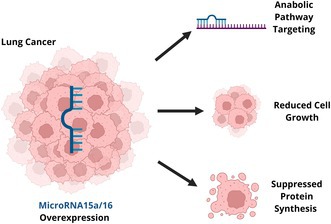

## 1 Introduction

Regulation of protein synthesis is a key component of cellular health, and nowhere are the consequences of dysregulated protein metabolism more evident than in non‐small cell lung cancer (NSCLC). While a range of metabolic reprogramming events are necessary to sustain NSCLC pathology [1], including increases in protein anabolism [2], a promising potential source of regulation for the cellular anabolic machinery can be found in the co‐transcribed microRNA species miR15a and miR16. MicroRNA (miRNA) are small strands of nucleotides which regulate the expression of potentially hundreds of genes by binding to the 3′ untranslated region (UTR) of mRNA transcripts and shepherding them for deletion in the RISC complex. Various miRNA are involved in numerous aspects of cancer pathology, including tumor development [3], progression [4], and metastasis [5], indicating that these small strands of nucleic acids may have a fundamental role as drivers of the cancerous phenotype. Independent investigations have documented that loss of miR15a and miR16 (hereafter referred to as miR15a/16) specifically encourages cell cycle progression [6] and that their rescue promotes radiosensitivity [7] in NSCLC, demonstrating the ability of these small nucleic acids to alter a variety of processes in lung cancer biology. However, their role in altering protein metabolism in NSCLC has not yet been determined.

Here we sought to investigate the role of miR15a/16 as regulators of protein anabolism in cancerous cells, with the hypothesis that overexpression of miR15a/16 would suppress cancer cell growth and protein anabolism in NSCLC. Previous studies have demonstrated that these key miRNA species have anti‐cancer activity in NSCLC [8, 9], but less work has been done in identifying their effect on tumor protein handling and cellular metabolism. Thus, we specifically hypothesized that overexpressing miR15a/16 in cellular models of NSCLC would suppress cell growth and protein synthesis.

## 2 Methods

### 2.1 Cell Culture

We employed two NSCLC cell lines, both purchased from ATCC (Manassas, VA, USA): H460 (ATCC Cat# HTB‐171, RRID:CVCL_0459) and A549 (ATCC Cat# CCL‐185, RRID:CVCL_0023). H460 cells were cultured in RPMI‐1640 (45000‐396, Corning Incorporated, Corning, NY, USA) and A549 cells in DMEM (11995040; Gibco, Grand Island, NY, USA). All media was supplemented with 10% fetal bovine serum (1500‐500; Avantor, Radnor, PA, USA), and 1% penicillin/streptomycin (Avantor), with cells housed in a humidified chamber maintained at 37°C and 5% CO_2_.

### 2.2 MiR15a/16 Overexpression

We transfected cell lines with miRNA mimics to miR15a‐5p (4464066‐MC10235) and miR16‐5p (4464066‐MC10339) purchased from ThermoFisher Scientific (Waltham, MA, USA), with negative control miRNA mimic (4464058) as control. We reverse transfected cells in 96‐well plates for proliferation experiments or 24‐well plates for protein synthesis, using Lipofectamine RNAiMAX (13778030; Thermo) in antibiotic‐free low serum transfection medium (Transfectagro, Corning 40‐300‐CV).

### 2.3 Cell Proliferation Assays

We measured cellular proliferation using a WST‐8 assay (HY‐K0301; MedChemExpress, Monmouth Junction, NJ, USA), a colorimetric assay that produces a tetrazolium salt in proportion to the number of cells in a sample well. For miRNA overexpression experiments, we added cells at a density of 10,000 cells/well to transfection agents (prepared using mimics plus Lipofectamine RNAiMAX (13778150; ThermoFisher) mixed in Transfectagro transfection medium (40‐300‐CV; Corning)) and allowed cells to attach overnight. The next day, we aspirated media from each well, and treated cells with either inhibitor or vehicle control at a concentration of 100 μM. From this point, every 24 h we refreshed wells with media containing 10% WST‐8 assay solution, per manufacturer's instructions. We incubated plates for 1 h, then the absorbance at 450 nm of each assayed well was measured using a plate reader with built‐in technical replication (Spectra Max I3x; Molecular Devices, San Jose, CA, USA). We repeated this process at 48 and 72 h using independent sets of experimental wells.

### 2.4 Protein Synthesis Measurements

We measured protein synthesis rates using deuterium oxide incorporation as described previously [10]. Briefly, we seeded plates at 50,000 cells/well, reverse transfected, replenished with normal media the next day, then exposed to deuterium oxide for 24 h. Following the incorporation period we retained a sample of media from each well and harvested cells in lysis buffer with proteins extracted in trichloroacetic acid (TCA). We determined the enrichment of cellular alanine (*E*_A_) using gas chromatography–mass spectrometry (GCMS, Agilent 7890a GC/5975c VL MSD, Santa Clara, CA, USA). We extracted free amino acid tracer from the reserved media, and calculated enrichment (*E*_CM_) via GCMS, then calculated protein synthesis rates using the equation $\frac{E_{A}}{E_{\mathrm{CM}}\times 3.7\times t\left(h\right)\;}\times 100$, where *E*_A_ represents amount of protein‐bound [^2^H]alanine (mole% excess), *E*_CM_ is the quantity of ^2^H_2_O in cell media (mole% excess), 3.7 represents the exchange of ^2^H between cell media and alanine (e.g., 3.7 of 4 carbon‐bound hydrogen of alanine exchange with water), and *t*(*h*) is the duration of tracer exposure measured in hours.

### 2.5 Bioinformatics Analysis

We used the DIANA‐mirRPath [11] web‐based analysis tool to investigate the associations between miR15a/16 and KEGG annotated molecular pathways. DIANA‐miRPath incorporates predicted interactions from TargetScan and data from experimentally supported miRNA targets in the TarBase database to provide predicted targets for specific miRNA. Our analysis included all genes that were targeted jointly by both miR15a and miR16.

### 2.6 Statistical Analysis

We assessed significant differences in protein synthesis rates using a two‐tailed *t*‐test, while we determined differences in proliferation measures by a two‐way ANOVA (group × time) with the Sidak correction used in the case of significant differences. For bioinformatics analysis, the DIANA software includes statistical procedures based on Fisher's Exact Test, utilizing a sampling algorithm which calculates a *p*‐value based on predicted KEGG pathway overlap. In this case, all values were FDR corrected. R [12] software was used for analysis of all experiments.

## 3 Results

### 3.1 MiR15a/16 Target Numerous Proteostatic Pathways

**FIGURE 1:**
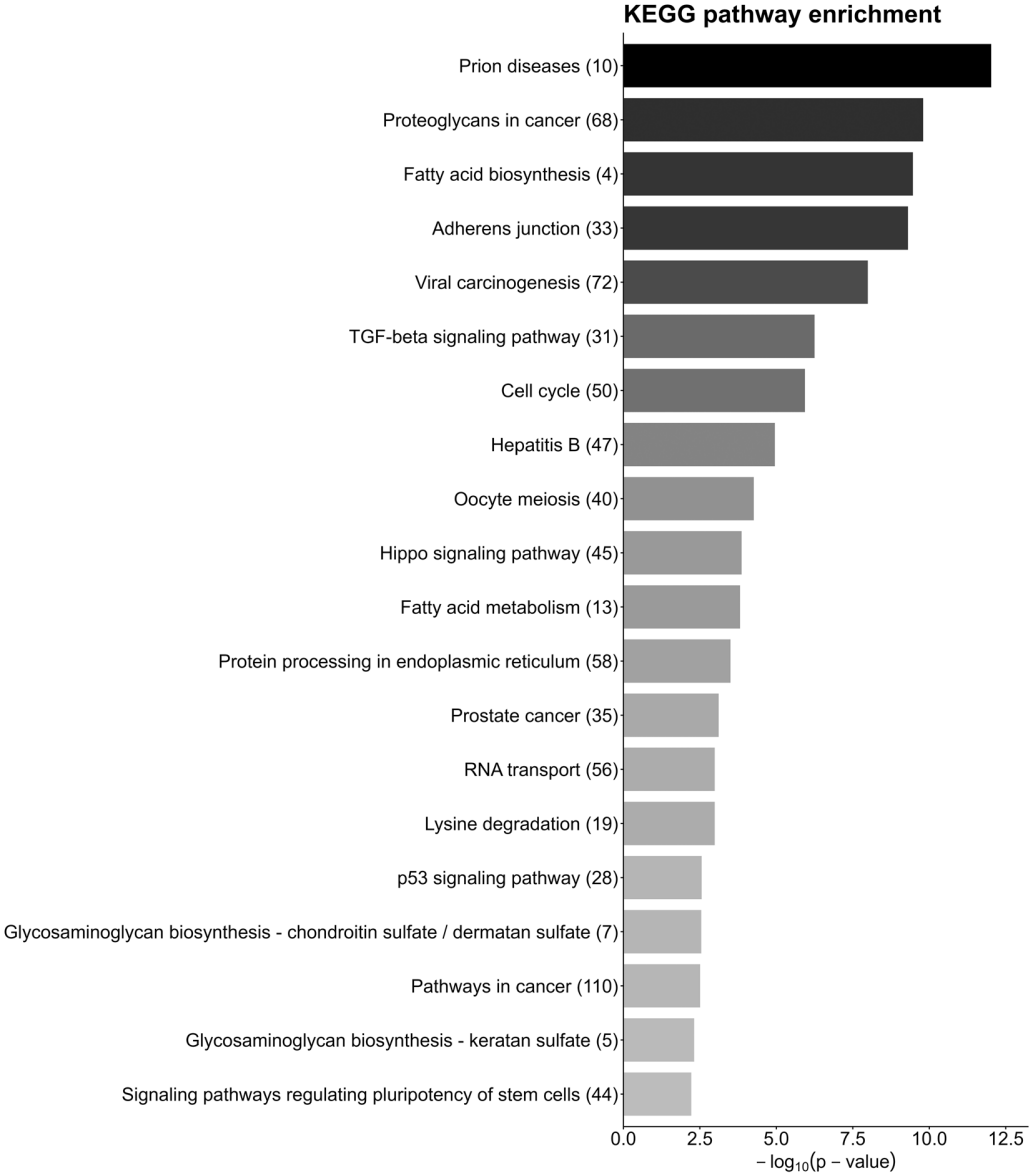
MiR15a/16 regulate multiple metabolic and cancer‐related pathways. Bar chart showing KEGG pathways targeted by miR15a/16, arranged by −log(*p* value), with the number of genes targeted in each pathway indicated in parentheses. *p* values were subject to FDR (Benjamini‐Hochberg) correction.

KEGG analysis of experientially verified miR15a/16 targets (Figure 1) revealed that these co‐transcribed miRNAs regulate genes are involved in numerous metabolic pathways and cancer‐related processes. This encompasses genes involved in cellular proteostasis, including ribosomal biogenesis, protein processing in the endoplasmic reticulum, TGF‐β signaling, p53 signaling, and ubiquitin proteolysis. Further, we found that mir15a/16 also regulate cancer‐specific pathways, including the cell cycle, proteoglycan metabolism, and TNF signaling, as well as specific genes involved in small‐cell lung, colorectal, thyroid, prostate, and bladder cancer.

### 3.2 MiR15a/16 Regulate Cell Proliferation and Protein Synthesis in NSCLC Cell Lines

**FIGURE 2:**
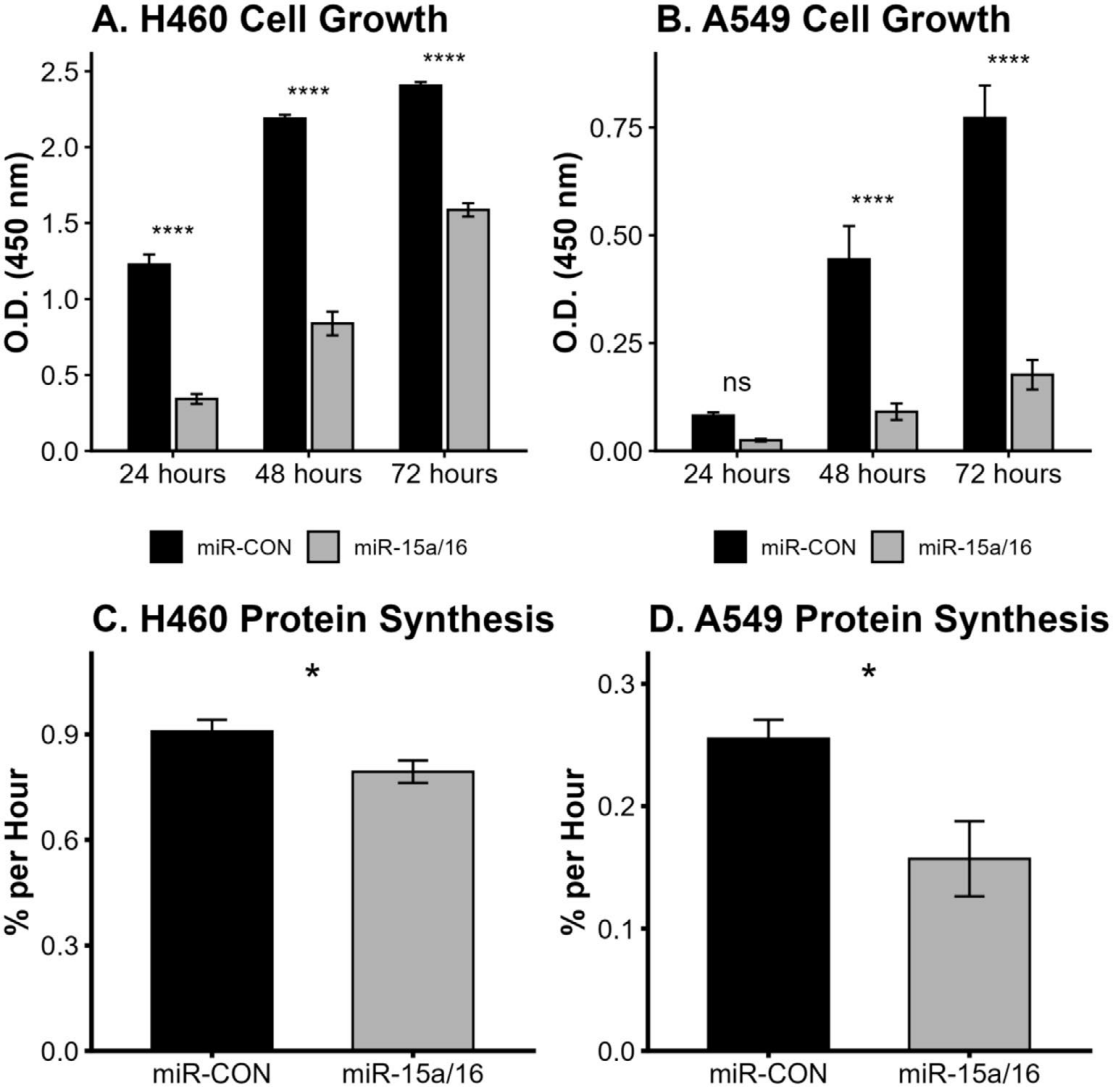
MiR15a/16 Suppress Cellular Growth and Protein Anabolism in NSCLC. Overexpression of miR15a/16 in H460 and A549 cells slows cellular proliferation over 72 hours (panel A & B) and blunts protein synthesis rates (panel C & D) in models of NSCLC. For proliferation experiments (panels A & B) *n* = 5, for protein synthesis measurements *n* = 4. * indicates a difference of *p* < 0.05, **** indicates a difference of *p* < 0.0001.

Overexpression of miR15a/16 suppressed cellular proliferation across 72 h (Figure 2) in both the H460 (24 h: −72.1%, 48 h: −61.6%, 72 h: −34.1%, all *p* < 0.05) and A549 (48 h: −79.5%, 72 h: −78.0%, all *p* < 0.05) cell lines. These miRNA also regulate protein metabolism in models of both types of NSCLC (Figure 2), suppressing protein synthesis rates by 12.6% in H460 cells (*p* < 0.05) and 38.4% in A549 cells (*p* < 0.05).

## 4 Discussion

Our data demonstrate that miR15a/16 regulate numerous genes in key metabolic signaling pathways, and that transfection of these miRNAs into cancerous cells slowed growth and suppressed protein synthesis in cellular models of NSCLC. Further, these experiments are the first to measure global protein synthesis in response to changes in miRNA, and indeed the first to show that miR15a/16 serve as regulators of protein anabolism in cancerous cells. It is worth noting that while we observed reductions in anabolism and growth in both cell lines, these reductions were greater in magnitude in A549 cells (a model of lung adenocarcinoma) than in the H460 cell line (a model of large cell lung cancer). This may be in part due to the differing base rates of anabolic activity in these cells, as H460 control cells displayed both greater proliferation and rates of protein synthesis compared to A549. Indeed, previous studies have found that large cell lung cancers grow faster than lung adenocarcinomas [13], indicating that there are fundamental metabolic differences between the two subtypes and hinting that miR15a/16 regulation of anabolism may have unique features depending on tumor etiology. Nevertheless, these coregulated miRNA suppressed growth in both models, indicating a common effect across lung cancer subtypes. We thus propose that miR15a/16 function as regulators of cellular proteostasis, capable of exerting simultaneous control over multiple cellular pathways regulating protein metabolism and potentially acting as tumor suppressors in NSCLC.

While our results are the first to demonstrate that miR15a/16 directly target cancer protein metabolism, they join others in showing that changes in the content of several miRNA species can serve as a trigger for shifts in the overall metabolic program of cancerous cells. Metabolic reprogramming describes the broad changes in cellular bioenergetics that are required to sustain growth in cancerous cells, and we documented numerous metabolic pathways containing genes that are regulated by miR15a/16. In this way, these two co‐transcribed miRNAs may exert broad effects on the metabolic state of the cancer cell, regulating not just protein‐handling processes but the biosynthesis of fatty acids and other biological compounds required to sustain shifts in cellular anabolism. This finding joins others in documenting the centrality of miRNA to the function of the cell, with various miRNAs regulating virtually every aspect of cell biology, highlighting their importance in understanding tumor pathophysiology.

More work remains to be done in identifying mechanisms by which miR15a/16, and miRNA more broadly, regulate cellular proteostasis and metabolism in cancerous cells. As such, future investigations into miR15a/16 in NSCLC will need to consider both the wide array of biological changes these compounds can produce, as well as the wide array of responses the cancer cell may make in response to them. Further, miRNA‐based therapies targeting cancer growth are in development using a number of mimetics against different tumor types [14], with several clinical trials of miRNA‐based treatments for cancer underway [15]. Considering these efforts, miR15a/16 mimetics present an exciting avenue for future anti‐cancer therapy development. While more work remains to be done in evaluating the role these compounds play in regulating tumor biology, these initial findings suggest that miR15a/16 are chief regulators of cell growth and proteostasis in NSCLC, potentially serving a role as tumor suppressors in this devastating disease.

## Author Contributions

P.J.R., P.P.N., and J.D.F. conceived and designed research; P.J.R. and B.C.G. performed experiments; M.J.G. and S.E.R. analyzed data; P.J.R. drafted manuscript; all authors read and approved the final version of the manuscript. Graphical abstract was created with BioRender.com.

## Funding

This work was funded in part by a Department of Defense Congressionally Directed Medical Program Award. Grant Number: W81XWH‐22‐1‐0988.

## Conflicts of Interest

The authors declare no conflicts of interest.

## Data Availability

All data generated during these experiments will be made available upon request to the corresponding author.
