# Correction to “MicroRNA 15a and 16 Regulate Proteostasis in Non‐Small Cell Lung Cancer”

**DOI:** 10.1096/fba.2026-00177

**Published:** 2026-06-11

P. J. Ryan , B. C. Guerra , P. Nghiem , S. E. Riechman , M. Janini Gomes , and J. D. Fluckey , “MicroRNA 15a and 16 Regulate Proteostasis in Non‐Small Cell Lung Cancer,” FASEB BioAdvance 8, no. 5 (2026): e70111, 10.1096/fba.2026-00075.PMC1313522342077822

The original author list included Peter Nghiem. The author's name should have read: Peter P. Nghiem.

The Results and Discussion sections referred to the H446 lung cancer cell line, instead of the H460 lung cancer cell line that was used in the study. Each reference to “446” should have read “460,” in reference to the H460 cell line that was actually used in the study.

Figure 2 caption inaccurately expressed controls as models of NSCLC. This caption should have read: “**FIGURE 2** | MiR15a/16 Suppress Cellular Growth and Protein Anabolism in NSCLC. Overexpression of miR15a/16 in H460 and A549 cells slows cellular proliferation over 72 hours (A, B, *n* = 5) and blunts protein synthesis rates (C, D, *n* = 4). * indicates a difference of *p* < 0.05, **** indicates a difference of *p* < 0.0001.”

The authors apologize for these errors.

